# Water abstraction affects abundance, size-structure and growth of two threatened cyprinid fishes

**DOI:** 10.1371/journal.pone.0175932

**Published:** 2017-04-17

**Authors:** Roberto Merciai, Carlota Molons-Sierra, Sergi Sabater, Emili García-Berthou

**Affiliations:** 1 GRECO, Institute of Aquatic Ecology, University of Girona, Girona, Catalonia, Spain; 2 Catalan Institute for Water Research (ICRA), Scientific and Technological Park of the University of Girona, Girona, Catalonia, Spain; Bournemouth University, UNITED KINGDOM

## Abstract

Hydrologic alteration is a major threat to freshwater biota, and particularly fish, in many river courses around the world. We analyzed and compared the effects of water abstraction on two threatened cyprinid fishes of contrasting ecology (the Mediterranean barbel *Barbus meridionalis* and the Catalan chub *Squalius laietanus*) in a Mediterranean stream. We compared abundance, size-structure, growth, and condition of both species across perennial and artificially intermittent reaches affected by water abstraction. Both species were less abundant, had scarce large individuals, and displayed slower growth rates (length-at-age) in intermittent reaches, showing clear detrimental effects of water diversion. Mixed-effect models of scale increments showed variation among individuals and among sites, years and age classes for both species. The larger-sized, water-column species (chub) disappeared or was rare in many intermittent reaches. The barbel present in intermittent reaches showed better somatic condition than in sites with permanent flow, perhaps due to reduced competition after rewetting or colonization by better fitted individuals. This benthic, rheophilic species seems more resilient to moderate water abstraction than chub. Many effects of water flow intermittency were only detected on fish life-history traits when accounting for natural, often non-linear, variation, along upstream-downstream gradients. Our results suggest that abundance was the strongest indicator of effects of water abstraction on fish populations, whereas condition was a more labile trait, rapidly recovering from anthropogenic disturbance.

## Introduction

Water scarcity is a main concern in intensively managed regions with low rainfall, where many small water courses often run partially or completely dry both because of lack of precipitations during extended periods of the year and water abstraction for human activities [1, 2]. In Mediterranean-climate areas, drought events have increased in intensity and frequency during the last decades and are expected to persist as a consequence of climate and land use changes [1, 3, 4].

When human activities increase the natural variability of flow interruption, physiological and behavioral adaptations of freshwater biota may no longer be effective to ensure the survival of populations to this kind of disturbance [5]. Habitat patches like pools, which usually provide refuge to aquatic life during the dry season, reduce their volume and disconnect to each other for longer times. Conditions become harsher in physical, chemical and biotic conditions, and extinctions at local scales may be common [6, 7].

Fish are among the taxonomic groups most affected by hydrologic alteration because they need larger water volumes, and have lower abundance and longer generation times than macroinvertebrates or algae [8, 9]. Mediterranean rivers support low fish richness, but most of their species are endemic and threatened according to IUCN criteria [10]. Modifications of Mediterranean fish assemblages (e.g. changes of relative abundance and decline of native and sensitive species) due to man-induced flow regime disruption have been well documented (e.g. [11–15]). Population and individual metrics may represent useful indicators of environmental perturbation in ecosystems with low species richness, such as Mediterranean streams [11, 16–19]. Less studies have been conducted about the consequences of flow reduction and intermittency on fish at the individual level, for instance on growth or condition [19], mostly focusing on salmonids (e.g. [20–23]).

Habitat quality and food availability mediate fish health, growth and somatic condition (e.g. weight-length relationship). Fish body condition is expected to be related with individual growth [24, 25] and population abundance (e.g. [26–28]). Growth and condition are known to be affected by physiological stress [29] and disturbances like drought and its associated environmental fluctuations (e.g. [17–21]).

The objective of our study is to examine the consequences of water abstraction on two threatened cyprinid species with different ecological niche in a small Iberian stream (Tordera Stream, NE Spain). Previous studies conducted in the same water course showed marked consequences of water abstraction on hydrology, thermal regime and fish assemblages [14, 30]. Here we analyze the effects on fish at population and individual levels, comparing abundance, size structure, growth and body condition between intermittent and perennial reaches. For growth, we also analyzed scale growth with mixed-effects linear models [31], in addition to length-at-age, to assess potential differences with respect to previous years. We expected to find lower fish densities due to summer mortality in sites impacted by severe flow intermittency (e.g. [13–15]), as well as lower growth rates and condition due to physiological stress and increased competition for food over summer, because of harsh physical-chemical conditions and overcrowding in refugia (e.g. [17, 20, 21, 32, 33]). We also expected lower sizes in intermittent sites, as a consequence of higher seasonal overall mortality [7] and size-selective mortality due to physiological stress (e.g. [34, 35]). We further hypothesized: i) that different fish attributes might vary in their degree of response to water abstraction, abundance being the slowest feature to recover, and individual condition the fastest; and ii) that effects of water abstraction are species-specific, with chub in general being more sensitive than barbel [36].

## Materials and methods

### Study area

The Tordera Stream rises in the Montseny Mountains (Catalonia, NE Spain) at about 1500 m a.s.l. and drains an area of *ca*. 895 km^2^. It is a typical Mediterranean stream with dry summers and occasional flash floods, the highest flows occurring in spring and autumn. Mean annual water yield is 170.4 million m^3^ year^-1^ and mean discharge is *ca*. 4 m^3^ s^-1^ [37]. Mean annual rainfall in the basin ranges from 1000 mm near the summit to 600 mm on the coast [37, 38]. There are no large dams along the stream. Land use in the Tordera catchment is agricultural, residential and secondarily industrial [39, 40]. The stream has a number of wastewater treatment plants (WWTP) mostly in the middle reach [41, 42]. Industrial and urban pollution were remarkable until the early 1990s [39] but the installation of WWTPs has improved water quality throughout the basin in the last decades. The effects of low summer flows have been exacerbated since the 1960s by increased legal and illegal water abstraction for irrigation and domestic use (about 34% of water yield) [37], leading to extensive dry beds affecting many kilometers of the stream, decrease of the aquifer level [14] and water temperature regime disruption [30].

The fish assemblage in the Tordera Stream is composed of both native and non-native species [14, 43]. The headwaters of the main Tordera and its tributary Arbúcies are dominated by stocked brown trout *Salmo trutta*, whereas in the middle and lower reaches, native Mediterranean barbel *Barbus meridionalis* and Catalan chub *Squalius laietanus* are prevalent, coexisting with eel *Anguilla anguilla* and introduced minnow *Phoxinus* sp. Mullets *Liza* sp. and non-native eastern mosquitofish *Gambusia holbrooki* are common in the lowermost reaches. Common carp *Cyprinus carpio*, largemouth bass *Micropterus salmoides* and other non-native species are occasionally captured in the watershed.

**Fig 1:**
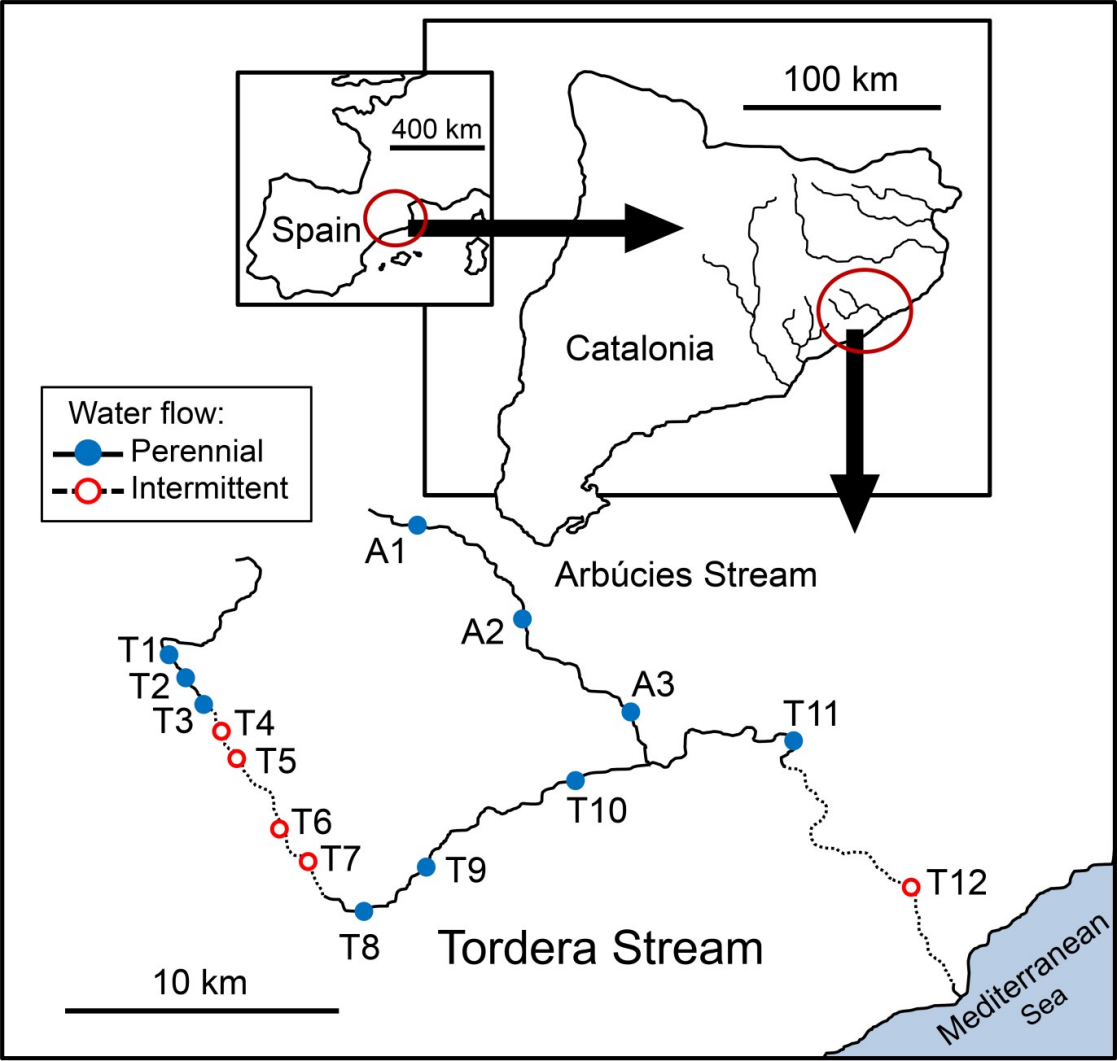
Location of the study area: Tordera Stream (T1‒T12) and its tributary, Arbúcies Stream (A1‒A3). The flow regime during the study period is also shown. See Table 1 for further details on the sampling sites.

Fish were sampled in fifteen sites, twelve on the Tordera mainstem (T1 –T12) and three on the Arbúcies tributary (A1–A3) (Fig 1, Table 1). Sites T1 to T3 had a relatively undisturbed habitat, with fast-flowing water, stony substrate, dense canopy cover, and relatively well preserved riparian zone. Site T3 was located immediately upstream of a weir, where a channel permanently diverts about 90 L s^-1^ for irrigation and urban use. In summer and early fall, most of the stream water is diverted into the channel and the downstream reach mostly dries or shows no discharge [14, 30]. Consequently, sites T4 to T7 were affected by seasonal dryness aggravated by water diversion, with a few, small isolated pools usually persisting in this reach during the driest months, fed by groundwater. These pools offer refuge to fish, that reach high densities until water flow resumes. From T8 to T11 the stream flows across several urbanized and industrial areas. In particular, most water flow in T8 in summer was constituted by the effluent of an urban wastewater treatment plant (WWTP) situated one km upstream, whereas T9 was located in the city of Sant Celoni. Site T12 was located in a reach also strongly impacted by diffuse water abstraction. These variations in water flow also affect the thermal regime along the Tordera course, especially in the intermittent reaches [30]. In these hydrologically-altered reaches, daily temperature variation is significantly higher and the relationship between air and water temperatures is weaker (see [30] for detailed analysis). Moreover, in T8 the WWTP input disrupts the natural daily temperature pattern [30]. Being located in a mountainous area, the Arbúcies tributary is much less affected by anthropogenic pressure, in comparison with the main basin of the Tordera. Complete dryness was never observed in sites A1 to A3 from 2004 to present, in contrast to sites T4‒T7 and T12 (Table 1).

**Table 1:** Physical and chemical features of the study sites at the Tordera Stream (sites T1‒T12) and one of its tributaries, Arbúcies Stream (sites A1‒A3).

Site	UTM (31T)	Altitude (m a.s.l.)	Wetted width (min‒max) (m)	Maximum depth (min‒max) (cm)	Conductivity (μs cm^-1^)	pH	O_2_ (mg L^-1^)	O2 (%)	Temperature (min‒ max) (°C)	Mean discharge (L s^-1^)	Flow regime
**Tordera**											
T1	450056, 4622372	340	5.24‒6.97	49‒66	105.24	7.87	9.64	94.73	-	149.3	Perennial
T2	450439, 4621726	319	6.17‒7.24	55‒73	110.03	7.88	9.77	95.27	-	309.5	Perennial
T3	450862, 4620754	305	4.95‒6.89	32‒53	109.27	7.64	9.64	93.60	12.33 (4.39‒19.56)	291.9	Perennial
T4	451946, 4619585	267	0‒5.83	0‒36	119.80	7.89	9.46	90.22	9.83 (4.07‒18.91)	167.7	Intermittent
T5	451666, 4619724	262	3.84‒7.36	25‒45	116.69	7.82	10.04	96.57	-	106.0	Intermittent
T6	452988, 4617886	213	0‒8.56	0‒37	113.24	7.42	9.79	95.38	-	152.5	Intermittent
T7	454803, 4615455	173	0‒7.09	0‒30	135.24	7.67	9.81	97.02	11.13 (7.14‒19.06)	217.0	Intermittent
T8	455681, 4614381	135	5.05‒11.94	32‒55	361.66	7.64	8.05	84.39	15.56 (7.25‒24.19)	212.7	Perennial
T9	459053, 4615859	120	6.02‒9.87	40‒62	457.71	7.74	8.49	86.90	15.11 (8.51‒20.59)	180.3	Perennial
T10	467054, 4619832	67	3.89‒13.90	19‒69	461.00	7.63	7.98	82.50	19.16 (12.85‒22.61)	-	Perennial
T11	474643, 4620999	36	7.25‒26.41	22‒62	514.50	8.13	9.24	97.35	20.60 (12.93‒24.13)	-	Perennial
T12	481311, 4611807	6	0‒49.05	0‒50	510.00	8.16	9.38	99.85	14.87 (6.46‒25.13)	-	Intermittent
**Arbúcies**											
A1	456886, 4630614	386	3.82–4.44	29‒41	245.57	8.21	9.55	94.37	-	-	Permanent
A2	462250, 4627821	212	4.21‒7.10	25‒44	296.27	8.03	9.54	93.87	13.80 (5.02‒22.3)	458268.8	Permanent
A3	467259, 4623025	91	2.54‒6.75	12‒41	310.33	8.24	9.84	94.37	-	-	Permanent

Average values of water properties during the study period (May 2012 –Oct 2013) are shown. Stream width and maximum depth were measured every 10 meters along 100-m transects. See Sampling and laboratory analyses for further details on the location and frequency of measurements.

### Studied fish species

The Mediterranean barbel *Barbus meridionalis* is a cyprinid fish endemic to the basins from Besòs (NE Spain) to Var (SE France) [44, 45]. It is a small-sized species, usually less than 270 mm standard length, with a clear benthic microhabitat. It is found in rather uncontaminated waters, feeds mainly on benthic invertebrates and the reproductive period may vary widely among different years, from March to July [46].

The Catalan chub *Squalius laietanus* was recently described by Doadrio, Kottelat and De Sostoa [47] as a separate species from *Squalius cephalus*. It is spread from the Ebro basin in Spain to the Agly River in France [45]. It is a medium-sized species, up to over 450 mm fork length [48] but usually smaller than 300 mm in the Tordera basin (García-Berthou et al., unpublished data). It is an omnivorous water-column dweller, found both in lentic and in lotic waters. Its spawning takes place between April and July [45, 46].

The two fish species have shown a contraction of their range in the last decades, due to water pollution, habitat degradation and loss of river continuity [12]. According to IUCN criteria, both species are included in the Spanish Red List as “Vulnerable” (VU) [45].

### Sampling and laboratory analyses

Scientific fishing permits were granted by the Catalan Government to two of the authors (RM and EGB) for sampling. The permits allowed the capture for scientific purpose of live fish of any species with no quantity restrictions, including threatened species, on the Catalan territory excluding protected areas. Electrofishing, netting and trapping were permitted. EGB has proved to the satisfaction of the Catalan Autonomous Ministry of Agriculture, Livestock, Fisheries, Food and Natural Environment that he meets the requirements necessary to carry out the tasks of scientist (person responsible for directing animal experiments) in accordance with the Catalan Government Decree 214/1997 of July 30th, regulating the use of animals for experimental and other scientific purposes. No fish were sacrificed for this study. All fish were captured by electrofishing, anesthetized to avoid unnecessary handling stress, and then released in the capture site after recovery in freshwater tanks. Therefore, no further permission is necessary from Ethics committees and research was conducted according to relevant national and international guidelines.

In each study site, a 100-m reach was sampled by one-pass electrofishing (Smith-Rooth LR-24 backpack electrofisher, 100–150 V, 0.8–1.5 A, fully rectified triphasic DC) every two months from May 2012 to October 2013. In this study, fish relative abundance was estimated as catch per unit effort (CPUE). In 10 of the 15 sites, fish were marked with Passive Integrated Transponder (PIT) tags and formed part of a detailed mark-recapture study, focusing on fish absolute abundance, survival, and capturability [49]. Four-pass removal with block nets in streams of this region indicated that: (i) 50–100% of the species and 40–60% of the individuals are generally captured with a single pass; and (ii) that the estimates of species richness and composition of a single pass are representative of the assemblage [50]. In the study stream, the mark-recapture study [49] shows that capture probability is generally lower (≤ 0.36) but absolute fish density and survival in the sites impacted by water abstraction is lower than in permanent sites, confirming the results we show below for CPUE. See the Discussion section for further comments on the adequacy of our sampling protocol to estimate relative abundance. A total of 1754 barbel and 765 chub were captured, anesthetized with 5–10 drops of clove oil (Aura Cacia^®^ pure essential oil) diluted in 10 L of water, measured to the nearest mm (fork length) and weighed to the nearest 0.1 g (S1 Table). Scale samples (5–10 each) of 860 barbel and 406 chub were collected from the same area above the lateral line and posterior to the dorsal fin. All fish were released in the capture site after recovery from electrofishing.

Concurrently with each fish sampling, we measured water conductivity, pH and O_2_ concentration using probes, and wetted width and maximum depth (every 10 meters along the 100-m transects). Water temperature was continuously measured at several sites using data loggers (HOBO Water Temp Pro v2, Onset Computer Cooperation, Boume, MA) and water flow was also measured hourly with calibrated data loggers (Heron Instruments dipperLog and Solinst^®^ 3001 Levelogger^®^ Edge, Ontario, Canada) in two of the sites. In the rest of sites, water flow was obtained from gauging stations from the Catalan Water Agency (data available at http://www.gencat.cat/aca). The temperature and flow data patterns have been analyzed elsewhere [30], where the exact location of the loggers is also given.

In the laboratory, fish scales were immersed for 30–60 minutes in a 5% KOH aqueous solution to eliminate skin remains, then mounted on glass slides and observed with a microfiche reader for age determination, discarding regenerated scales [51]. An annulus was taken as a transition between two uninterrupted zones of closely- and widely-spaced circuli. In the case of chub an essential criterion for annulus identification was anastomosis, namely several circuli which cut across (cutting-over) several others on the rest of the scale [52]. To estimate ageing precision, a subsample of 50 scales for each species was examined independently by two researchers. Radius length was measured on the oral side of the scale for every annulus recognized, to determine the annual fish scale size increment (S2 Table), i.e. the distance between two annuli on the fish scale [53].

### Statistical analyses

The main statistical analyses to test for the effects of flow regime (FR, hereafter) on all response variables consisted in building minimum adequate models (MAM) with linear models. A MAM is defined as the model that contains the minimum number of predictors that satisfy a given criterion, for example, the model that only contains predictors that are significant at some pre-specified probability level [54]. We obtained MAMs for the linear models of response variables (site averages for density, lengths, condition, growth (size-at-age), and scale increment) using altitude as a covariate, and FR (perennial vs. intermittent) as a categorical factor. The initial models are identical to analyses of covariance (ANCOVAs) but included a quadratic component of altitude, to account for nonlinear natural variation along the stream (see e.g. [55, 56] for a similar approach), and interactions between covariates and flow regime. We obtained the MAMs by removing all non-significant interactions and main effects (*P* > 0.1). We used FR as a binary categorical factor because: i) although diffuse water abstraction through wells also occurs in the basin, the presence of the weir and diversion channel creates a severe, local hydrological perturbation that effectively turns off the intermediate stream segment into an intermittent stream, in contrast to most of the mainstem; ii) previous studies have already shown significant differences between the fish assemblages of the intermittent and permanent reaches [14]; a single continuous descriptor (e.g. percent of water abstracted) is difficult to obtain due to unquantified diffuse water abstraction.

To analyze fish condition (weight-length relationship), we used ANCOVAs of weight as the response variable, sampling site and season (spring, summer or fall) as categorical factors, and FL as the covariate. FL and weight were log_10_-transformed, to satisfy the statistical assumptions (normality, homoscedasticity, and linearity). ANCOVAs were also used to compare fish growth among different sampling sites and seasons (categorical factors), with fork length (FL) as dependent variable and age as covariate. All ANCOVA models included interactions among covariates and categorical factors, which test the equal slopes assumption of standard ANCOVA, and were only removed if they were non-significant (*P* > 0.1), following García-Berthou and Moreno-Amich [57]. A more liberal significance level (*α =* 0.1) was used as recommended by Huitema [58] because a non-significant result is not evidence for the null hypothesis. Estimated marginal means of the dependent variables are the means for each level of the factor, adjusted for covariates with ANCOVA, and were used to describe the differences in fish condition and growth across sites [57] and were also used as response variables to obtain the abovementioned MAMs (S3 Table). These ANCOVAs were performed with SPSS 20.

We used Bowker’s test of symmetry [59] and *t*-tests to check for systematic bias between the age estimations of the two agers, using the functions “ageBias” and “agePrecision”, respectively, of the “FSA” package [60] of the R environment [61]. Bowker’s (Hoenig's) test for symmetry tests the null hypothesis that the frequencies of age estimations are symmetric between the two agers (i.e. there is no systematic bias) [59‒60]. We also computed the coefficient of variation (CV) of age readings as a measure of precision by using the same package.

The effects of age, site and year on fish scale increment were estimated with mixed-effects linear models (MELMs), following Weisberg and co-authors [31, 62]. This method has fewer assumptions than others based on fish length back-calculation, analyzing the annual fish scale size increment (dependent variable) with no need of a particular relationship between scale radius and fish size. Age was considered as a fixed-effect factor, whereas site, year of growth, fish individual and their interactions were treated as random-effects factors. Analyses were performed using the function “lmer” of “lme4” R package [63] and the function “rand” of “lmerTest” package [64]. Moreover, we used Pearson’s correlations to test for relationships between the contribution of the year of growth to scale size annual increment and climatic features (yearly averages of air temperature, precipitation, stream flow, proportion of days of drought per year) measured at various sites in the watershed (Data from ACA: https://aca-web.gencat.cat/aca/appmanager/aca/aca/) starting from 2004, year in which the oldest fish found were born.

## Results

**Fig 2:**
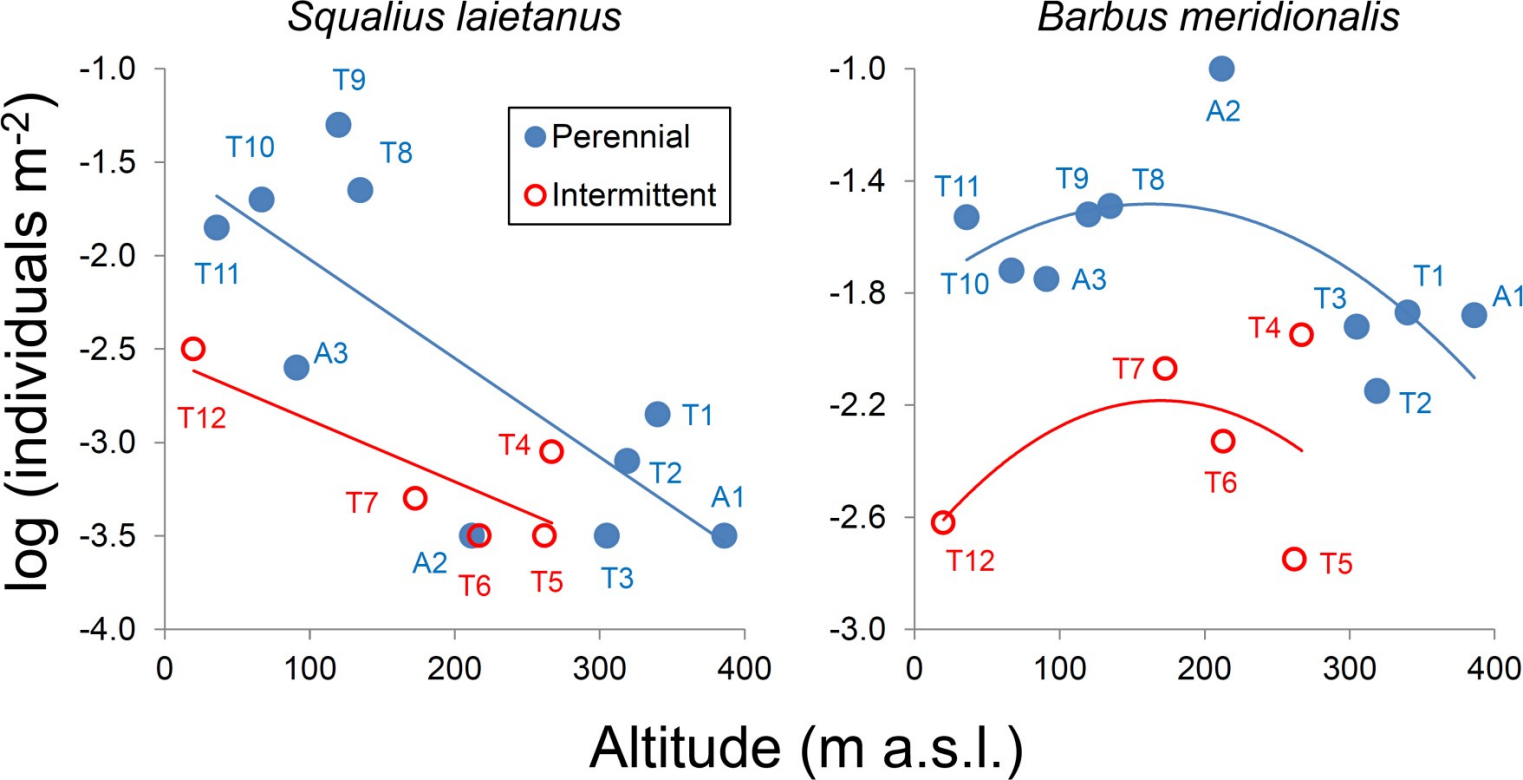
Relationship of mean fish abundance (May 2012‒October 2013) with altitude by flow regime and fish species. The lines correspond to linear (*S*. *laietanus*, top plot) or quadratic regressions (*B*. *meridionalis*, bottom plot) separated by flow regime. Linear *r*^2^ are 0.668 (perennial) and 0.620 (intermittent). Quadratic $R_{adj}^{2}$ are 0.407 (perennial) and 0.223 (intermittent). Sites T1‒T12 are located on Tordera mainstem; sites A1‒A3 are on the Arbúcies tributary. *Squalius laietanus* was not captured at sites T3, T5, T6, A1, and A2.

Many response variables displayed nonlinear variation along the upstream-downstream gradient, probably due to natural ecological optima, and this was considered in order to test the effects of flow regime (Table 2). For instance, barbel density showed a unimodal response, peaking in the middle course of both Tordera and Arbúcies streams (Fig 2). By contrast, chub density fitted to a linear model showing higher values in the middle and low course of Tordera mainstem, with scarce or no appearance in other sites (Fig 2). The effect of water intermittency was highly significant for the two species after accounting for the variation with altitude, with ca. 60% of explained variation (*R*^2^_adj_) for both models (Table 2). The two species were less abundant in the intermittent reaches. Barbel densities (mean CPUE ± SE) were 53.9 ± 17.3 in intermittent and 280 ± 88.7 fish ha^-1^ in perennial reaches. Chub densities were 7.24 ± 5.5 in the intermittent and 111 ± 53.5 fish ha^-1^ in the perennial reaches.

**Table 2:** Minimum Adequate Models (MAMs) of six population and individual features (response variables; see text for details) of *S*. *laietanus* and *B*. *meridionalis* in the Tordera basin in 2012 and 2013.

Response variable	MAM	*P* (FR)	*R*^2^_adj_
***S*. *laietanus***			
**Density**	FR + Alt.	0.001	0.600
**Mean FL**	FR × Alt. × Alt.^2^	0.065	0.916
**Maximum FL**	‒	‒	‒
**Condition**	‒	‒	‒
**Growth**	‒	‒	‒
**Scale Increment**	‒	‒	‒
***B*. *meridionalis***			
**Density**	FR + Alt. + Alt.^2^	0.001	0.604
**Mean FL**	‒	‒	‒
**Maximum FL**	FR × Alt. × Alt.^2^	0.005	0.844
**Condition**	‒	‒	‒
**Growth**	FR × Alt. × Alt.^2^	0.014	0.416
**Scale Increment**	FR × Alt. × Alt.^2^	0.014	0.601

The linear models tested the effect of Flow Regime (FR) (categorical factor) controlling for altitude (Alt.) (linear and quadratic effects). Terms that showed no significant effects (*P* > 0.1) were removed from the model. “‒” indicates that no significant terms were identified. The *P* value corresponds to the effects in the selected model of the flow regime (perennial vs. intermittent). The response variable for condition corresponds to the estimated marginal means of the analysis in Table 3.

**Fig 3:**
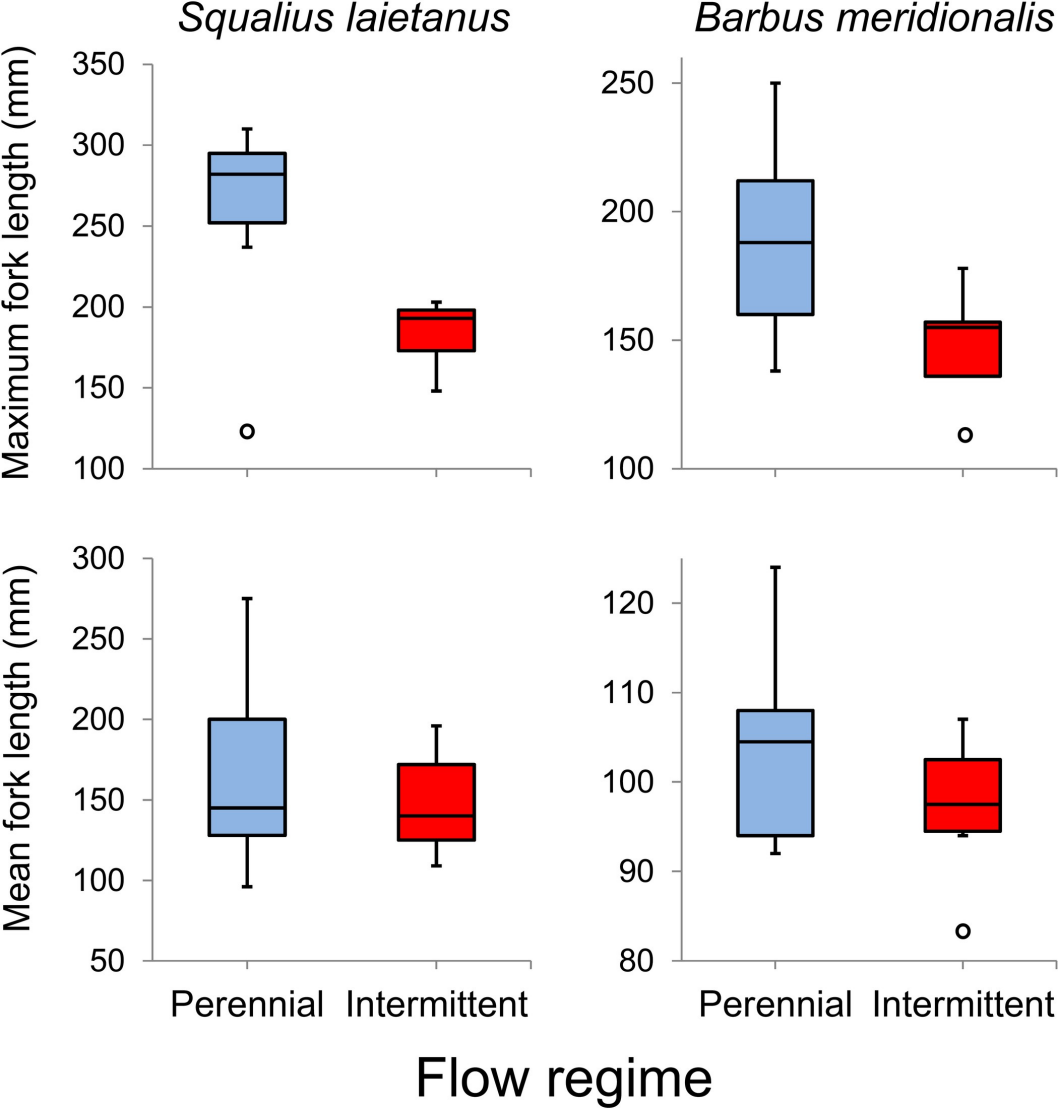
Maximum and mean size of fish captured in the Tordera basin during 2012 and 2013, comparing perennial and intermittent reaches. Boxes represent the first and third quartiles, lines are the medians, and bars are maximum and minimum values, excluding outliers (circles).

Quadratic models of altitude with interacting effects of flow regime were selected for mean length of chub and maximum length of barbel, both explaining high percentages of variation (> 84%) and with observed lengths lower for both species in the intermittent reaches (Fig 3).

**Fig 4:**
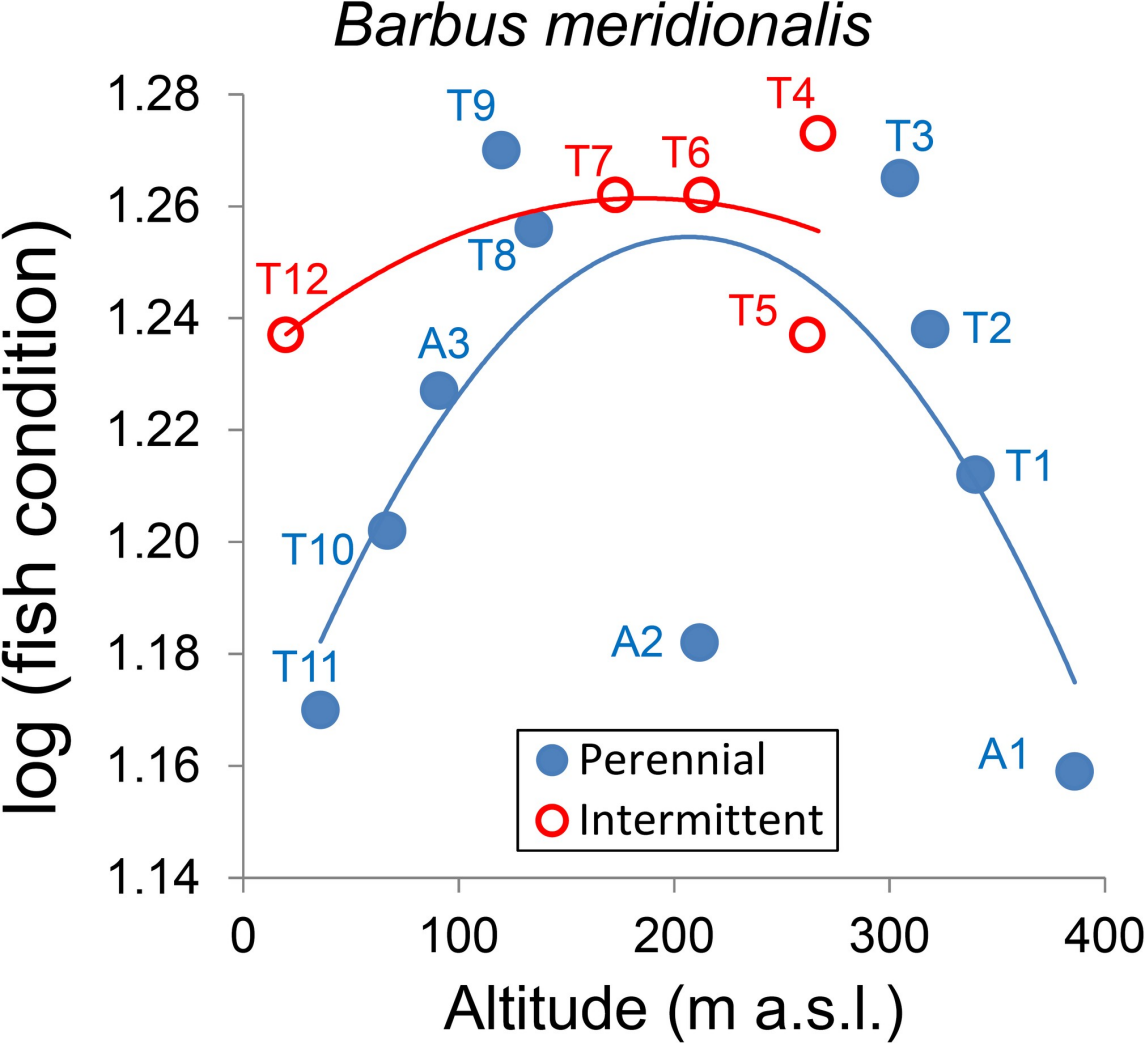
Relationship of fish condition (total weight adjusted for fork length with analysis of covariance) of *B*. *meridionalis* with altitude. The lines correspond to quadratic regressions by water flow regime. Total weight and fork length were log-transformed. Quadratic $R_{adj}^{2}$ was 0.406 for perennial reaches and 0.375 for the intermittent reaches.

**Fig 5:**
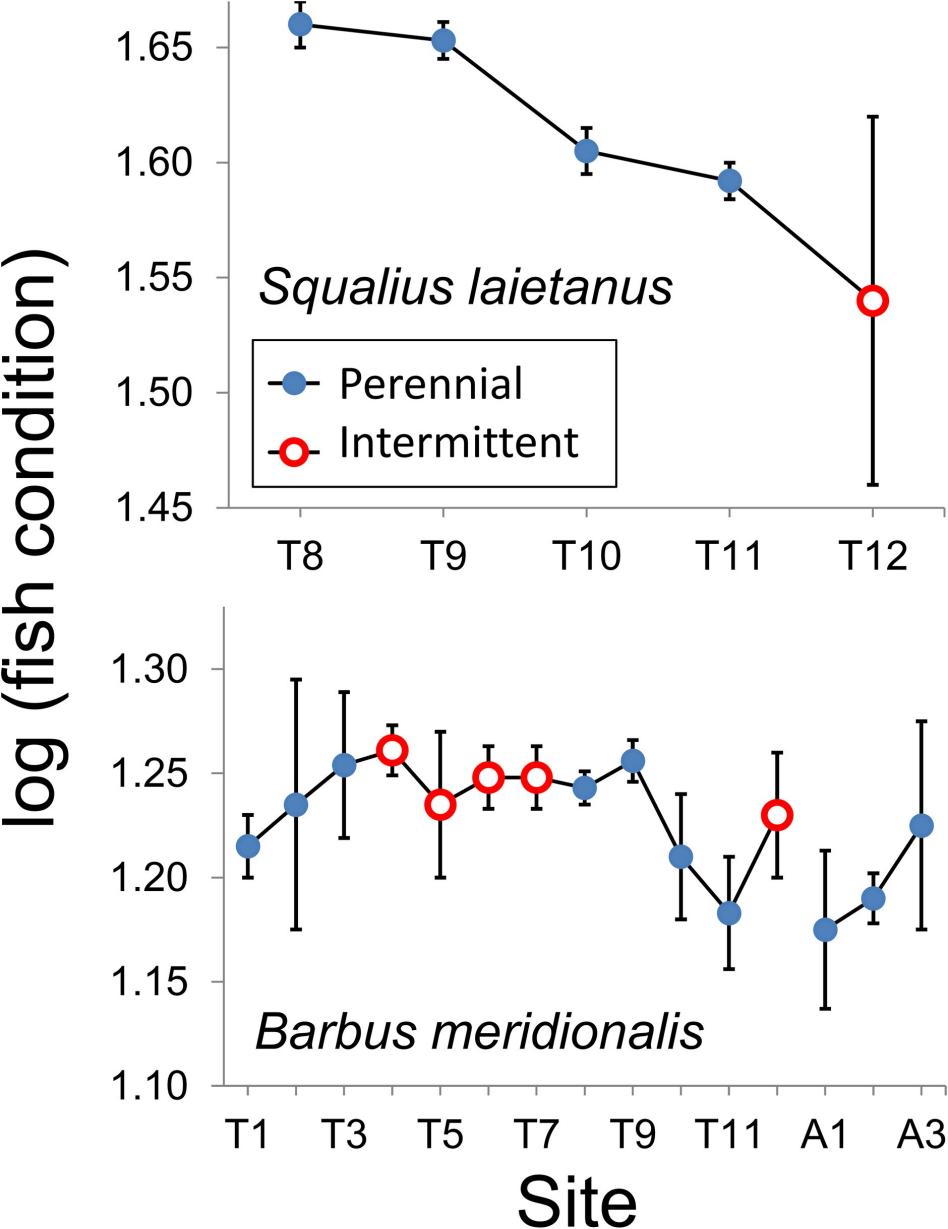
Marginal means of fish condition (total weight adjusted for fork length with analysis of covariance) across sampling sites (Table 2). The mean log_10_ of fork length (at which fish total weight was adjusted) was 2.165 for chub and 2.01 for barbel. Non-estimable means are not shown. Sites T1‒T11 are located on Tordera mainstem and sites A1‒A3 are on the Arbúcies tributary.

Both species displayed among-site variation in fish condition, with significant site × length interaction indicating different slopes of the weight-length relationship (Table 3). Barbel condition displayed a quadratic variation with altitude (Fig 4) similar to abundance and was marginally higher in the intermittent than in perennial reaches (*P* = 0.07, *t*-test) (Figs 4 and 5).

**Table 3:** Analyses of covariance of condition (top) and growth (bottom) of chub and barbel captured in the Tordera basin (May 2012‒October 2013).

	***S*. *laietanus***				***B*. *meridionalis***			
	***R***^**2**^_**adj**_ **= 0.988 **				***R***^**2**^_**adj**_ **= 0.978**			
**Total weight**	**SS**	**df**	***F***	***P***	**SS**	**df**	***F***	***P***
**Fork length (FL)**	22.95	1	6667.0	< 0.0005	15.94	1	5071.0	< 0.0005
**Site**	0.117	3	11.34	< 0.0005	0.096	14	2.173	0.007
**Season**	0.144	5	8.398	< 0.0005	0.016	2	2.510	0.082
**Site × FL**	0.124	3	12.00	< 0.0005	0.076	14	1.729	0.045
**Season × FL**	0.155	5	9.007	< 0.0005	0.010	2	1.610	0.200
**Site × Season**	0.180	9	5.807	< 0.0005	0.005	3	0.576	0.631
**Site × Season × FL**	0.181	9	5.853	< 0.0005	0.004	3	0.421	0.738
**Error**	1.552	451			2.556	813		
	***R***^**2**^_**adj**_ **= 0.596 **				***R***^**2**^_**adj**_ **= 0.686**			
**Fork length**	**SS**	**df**	***F***	***P***	**SS**	**df**	***F***	***P***
**Age**	1.445	1	181.6	< 0.0005	0.734	1	156.9	< 0.0005
**Season**	0.012	2	0.742	0.477	0.031	2	3.284	0.038
**Site**	0.120	4	0.005	0.005	0.417	14	6.372	< 0.0005
**Season × Site**	0.006	2	0.352	0.703	0.083	3	5.929	0.001
**Season × Age**	0.017	2	0.017	0.336	0.015	2	1.591	0.204
**Site × Age**	0.125	4	3.943	0.004	0.351	14	5.364	< 0.0005
**Season × Site × Age**	0.014	2	0.904	0.406	0.156	3	11.15	< 0.0005
**Error**	2.760	347			3.816	816		

The first analysis tested differences of total weight (dependent variable) across sites and seasons (categorical factors), and their interactions, controlling for fork length (covariate). The second analysis tested the effects of sampling season and site (categorical factors), and their interactions, on fork length (dependent variable) controlling for age (covariate). Total weight and fork length were log-transformed. SS = sum of squares, df = degrees of freedom.

Similarly than for abundance, chub condition was highest in T8‒T9 and progressively decreased along the stream, from the middle reaches to the lowermost and intermittent reach (Fig 5). Chub condition displayed significant among-site and seasonal variation and also site × length interaction.

**Fig 6:**
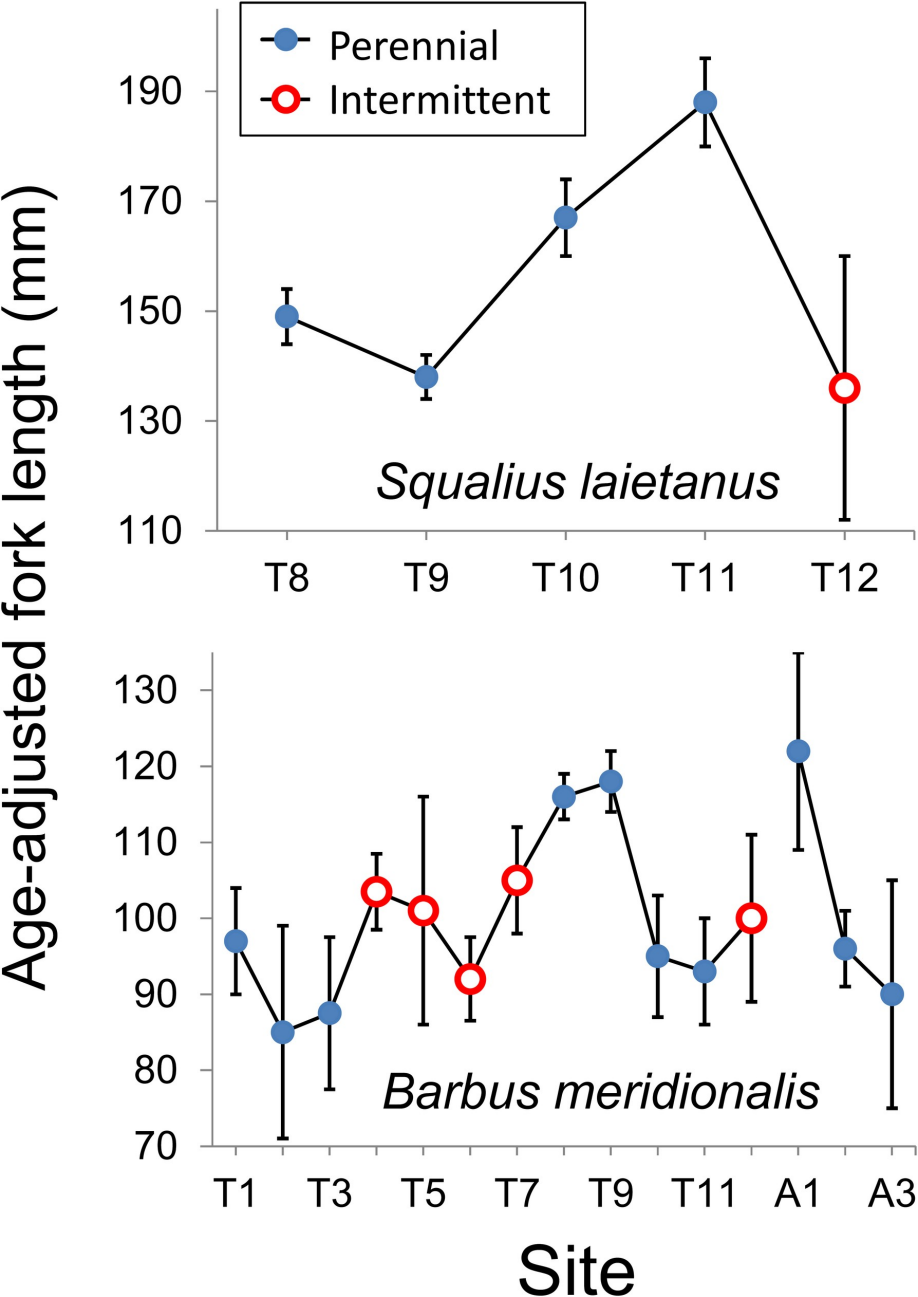
Marginal means of fish fork length (adjusted for fish age with analysis of covariance) across sampling sites (Table 2, below). The mean age (at which fish size was adjusted) was 2.82 years for *S*. *laietanus* and 2.83 years for *B*. *meridionalis*. Non-estimable means are not shown. Sites T1‒T11 are located on Tordera mainstem; sites A1‒A3 are on the Arbúcies tributary.

Although age estimation varied moderately between the two readers (CV of 9.7% for barbel and 7.9% for chub), there were no significant asymmetries in the age readings of barbel (Bowker’s test: *χ*^2^ = 8.8; df = 4; *P* = 0.066) and chub age (*χ*^2^ = 6.3; df = 6; *P* = 0.39) (S4 Table) and no systematic biases (*t*-tests, *P* > 0.3) (S1 Fig). Both species also showed significant variation in growth (length-at-age) among sites and with site × length interaction (Table 3). Barbel growth (length-at-age) also showed quadratic variation with altitude, interacting with flow regime (Table 2), and slightly lower age-adjusted fork length in intermittent reaches. The intermediate reaches T8 and T9 showed high rates of barbel growth (Fig 6), in agreement with the results of density and condition. Chub growth was higher in permanent reaches that in the intermittent reach, where it was more abundant (Fig 6).

**Fig 7:**
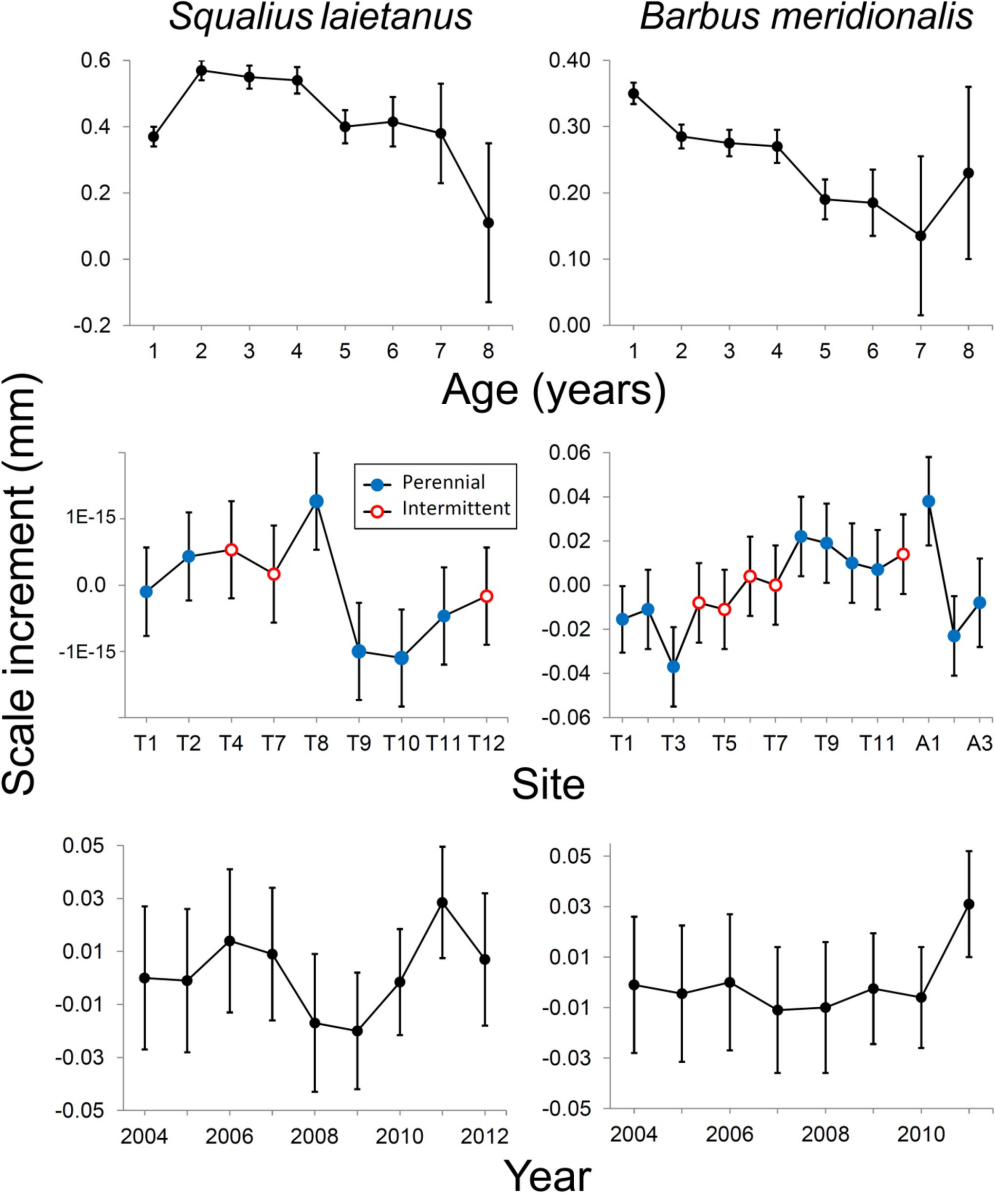
Effects of fish age, sampling site and year of growth on yearly increments of fish scale oral radius, as estimated by the mixed-effects models. See Table 3 and text for significance of the factors. The bars how standard errors. Codes of sampling sites as in Table 1.

Barbel annual scale increment was significantly affected by flow regime, after accounting for the nonlinear upstream-downstream variation (Table 2). The average increment of scales for barbel showed significant age × site × year, age and individual effects (Table 4) and was slightly higher in reaches with perennial water flow (0.0004 *vs*. -0.0008 mm). Scale increment showed a general upstream-downstream increase upstream-downstream trend in the Tordera mainstem (Fig 7). In the case of chub, age × site × year and individuals effects were also significant (Table 4) and scale increment was slightly higher in intermittent reaches.

**Table 4:** Mixed-effects linear models of size increments in fish scales collected between May 2012 and October 2013 for chub (*Squalius laietanus*) and barbel (*Barbus meridionalis*).

	*Squalius laietanus*			*Barbus meridionalis*		
Random effects	*χ*^2^	df	*P*	*χ*^2^	df	*P*
**Year**	1.830	1	0.180	1.763	1	0.184
**Site**	0.000	1	1.000	4.406	1	0.036
**Year × site**	0.000	1	1.000	2.836	1	0.092
**Age × site**	0.000	1	1.000	0.849	1	0.357
**Year × age**	0.000	1	1.000	2.588	1	0.108
**Age × site × year**	5.770	1	0.020	10.02	1	0.002
**Fish individual**	3.920	1	0.050	17.74	1	< 0.0005

Fish age was treated as a fixed-effect factor, whereas year of growth, sampling site, fish individual, and interactions were treated as random effects.

For the two species, age × site × year and individual were the most important sources of variation (Table 4). The youngest age classes (1 and 2 year old) had the highest average growth rates, which declined with age. Environmental factors seemed favorable to barbel growth in 2012, whereas the best growth year for chub was 2011, after three years of slowest growth (Fig 7). Barbel annual size increment was positively correlated to the proportion of days with discharge equal to zero recorded in T9 (Pearson’s *r* = 0.773; *P* = 0.024), whereas a marginally significant correlation (*r* = 0.602; *P* = 0.082) was found between chub increment and air temperature measured in St. Celoni, close to T9.

## Discussion

### Effects of water abstraction

The density of the two species was noticeably higher in the perennial reaches. Chub was the species most affected by water abstraction, and this caused its disappearance from a long intermittent reach where it was present historically [14, 43]. However, more significant effects of water abstraction for other variables were obtained for barbel because it is more widespread in the basin, whereas chub is only present in some stream segments. Several cases of flow reduction have been associated to decreased fish abundance (e.g. [15, 65, 66]), as well as recent fish population contractions or local extinctions related to hydrologic alteration in the Tordera catchment [14, 67–69]. Considerable numbers of dead fish of several species (including the two study species as well as brown trout and *Phoxinus* sp.) were directly observed during the study period, mostly in reaches immediately after pool drying. On the other hand, high densities of barbel, with occasional presence of chub, were detected in pools persisting during summer, suggesting the existence of a drought-escape behavior for these fishes. This behavior has been described in regions affected by dry summers, where the initial phase of drying may promote fish movement [70–72]. These observations allowed us to recognize streambed dryness as a leading cause of low fish density in these reaches, associated to death and migration [13, 73].

Natural variation in fish longitudinal distribution can in part explain the observed pattern of barbel and chub survival, since lower reaches have in general more stable ecological conditions, are nutrient-rich and show higher water temperatures than the uppermost ones [74, 75]. In particular, the middle reaches showed the highest fish densities, probably because of both perennial flow regime but also because the higher nutrient input increases ecosystem productivity. Chub, moreover, is known to colonize preferentially deep pools and runs [45], and riffles are the most common habitat in the upper course of Tordera and Arbúcies, where few individuals of this species were found.

We are confident that the effects of water abstraction on fish abundance are real and not due sampling issues for a number of reasons. Although the two lowermost sites had larger wetted widths (but depths much less that the maximum depth in most of channel) during one of the samplings (during a high flow), 13 out of 15 sampling sites had widths much less than 15 m, and thus the European Standard on electrofishing (EN 14011), which requires a minimum length of 50 m to assess fish abundance and age structure, was always followed. The length of 100 m that we used everywhere might have been less adequate to estimate fish richness and species composition in a few occasions [50] but should not affect individual level metrics, such as condition or individual growth. In all sites, depth was always less than 75 cm (often much less) and thus electrofishing efficiency was high. More importantly, a concurrent mark-recapture study, which is arguably the best method available for this purpose, confirmed the effects of water abstraction on fish abundance [49].

### Effects of water intermittency at individual level

Our observations confirmed the presence of smaller individuals of barbel and chub in the Tordera intermittent reaches [14]. This pattern may be due to three possible factors: i) high mortality rates in intermittent sites, that imply lower probabilities for fish to live many years and attain large sizes; ii) higher mobility of larger individuals, potentially more capable to move away from impacted reaches [34, 72]; and iii) size-selective mortality due to physiological stress, which is known from *Barbus*, *Squalius* and other Mediterranean cyprinid species as a consequence of flow intermittency [34, 35, 76, 77] because of higher oxygen demand of larger individuals [78, 79].

Fish condition and growth were expected to vary across sites and seasons, and to be lower in the intermittent reaches. Such a response to low flow conditions has been reported for barbel *Luciobarbus* sp. (e.g. [17, 32, 33]), salmonids (e.g. [20, 79]), and smallmouth bass [80]. Our observations partially confirmed our hypothesis. Growth, measured as length-at-age, was lower at intermittent reaches for both chub and barbel. Scale increments showed slightly different results (lower for barbel but higher for chub at intermittent reaches), possibly because increments in calcified structures are not always well correlated with growth rates [81] and because the models used for scale increments average increases in many growing seasons and not final size-at-age and account for many other factors (e.g. year and individual). Surprisingly, barbel from intermittent reaches showed better condition (weight-length relationship), comparable with the ones from the urbanized reaches in the middle Tordera course, in which fish of the two species looked healthy and abundant. Another study [25] reports both higher condition and growth of cyprinids in sites affected by summer dryness. Barbel populations from permanent streams, moreover, have been reported to show a slender body profile and lower condition than populations from intermittent streams, a likely adaptation to higher flow and water velocity [18]. Fish inhabiting instable environments like intermittent streams may require high levels of energy reserves, i.e. high body condition, a likely investment to increase reproductive success [18, 19, 82, 83]. These independent evidences cannot exclude that high barbel condition in the Tordera intermittent reaches may also depend on adaptive phenotypic plasticity. In several other cases, by contrast, barbel condition was reported to be positively correlated to riparian vegetation cover, water flow and related environmental variables, such as conductivity and oxygen concentration (e.g. [16, 17, 32, 33, 84]). Mas-Martí et al. [17] attributed the lower condition of *B*. *meridionalis* and *S*. *laietanus* in an intermittent tributary of the Tordera to two main factors, namely the lower temperature and productivity of the tributary, and bottom-up effects of stream dryness on the trophic web, leading to lower food availability for fish. In our case, part of the upper intermittent reach was covered by a dense tree canopy, which coupled with hyporheic flow kept temperature and dissolved oxygen within tolerable levels, mitigating the physiological stress for fish. Moreover, strong competition for food was limited to some weeks in the summer refugia, but high invertebrate availability per capita could be guaranteed in the other seasons for the few fish that survived drying. Alternatively, fish with better condition might be more capable of recolonizing intermittent reaches. Overall, dryness increases mortality and decreases abundance and growth but fish present may show better individual condition due to better colonization capability or reduced resource competition.

Chub was usually present, at low densities, in only one of the intermittent sampling reaches, the lowermost one, making difficult to compare traits among impacted and control sites. Our results show a gradual condition decrease from the most urbanized sites to the lowermost, intermittent one, rather than a simple distinction between sites with different flow regimes. In fact, most life-history traits showed variation, often non-linear, with altitude, which had to be accounted to adequately test the effects of flow regime. Density, growth, and condition of barbel were highest at intermediate altitudes. These patterns have been observed in many freshwater fishes but might also be due in part to anthropogenic factors. The middle reaches of the Tordera mainstem are a nutrient-rich zone affected by WWTP effluents [41, 42]. These findings contrast with those of Britton et al. [84], who found higher *Barbus* growth rates in presence of low phosphate loads, but match observations with other European cyprinids such as *Rutilus rutilus* (e.g. [85, 86]). Arguably, a moderate nutrient enrichment in the middle Tordera course may increase barbel growth and condition if they do not exceed the tolerance limits of this species. In agreement, age at length of barbel and scale increments for both species were high in the more eutrophic site T8.

The growth-at-age of the two species may reflect sexual maturation. This occurs at age 1 for most individuals of barbel, whereas in chub it is more variable [46] and may correspond to the first growth decrease observed after the second year of life [87, 88]. Furthermore, as chub is a multiple spawner with reproductive season potentially protracted [46] we cannot exclude that a significant fraction of the newborns hatch at the beginning of an unfavorable period, resulting in low growth rates in the first year [88–91]. Chub size increment was higher in warmer years: this was expectable, since temperature is a dominant factor, together with food availability, in determining fish growth patterns in space and time (e.g. [87, 88]).

Our research confirmed the existence of strong effects of hydrologic alteration at the population and individual levels for the two study species. Population and individual metrics showed highest values in the middle Tordera course, where flow is permanent and nutrient availability is higher due to WWTP inputs. Body condition was not negatively affected by stream drying, revealing how individual traits vary in the potential to inform about hydrological regime disruption. Finally, it is important to stress how the effects of drying were often highlighted only after controlling for natural variability through appropriate statistical tools. Chub was the species most affected by drought, being absent from most impacted sites. Barbel seemed better adapted to take advantage of moderate environmental disturbance, probably through increased resistance and colonization ability, except in extreme situations in which it is wiped away. We would expect this species to recover quickly in terms of abundance in the intermittent reaches if drought events were less intense and lasting, whereas chub was revealed as more sensitive to reduced stream flow and less resilient to hydrologic alteration.

## Supporting information

S1 Table Fork length and total weight of all fish captured during the study period (May 2012 –October 2013). (XLSX)

S2 Table Estimated age and scale annual radium length of fish used for annual size increment analyses. (XLSX)

S3 Table Values of the response variables used for Mininum Adequate Models. (XLSX)

S4 Table Data for the Bowker’s symmetry tests for the age estimations of chub (*Squalius laietanus*) and barbel (*Barbus meridionalis*) by two of the authors. The values on the diagonal are scale samples with the same age estimation for both readers. See text for statistical results.
(DOCX)

S1 Fig Between-reader differences in age estimation (mean and SEs are shown). Numbers above error bars are the number of fish.
(PDF)
