# Supplementary material for: Water abstraction affects abundance, size-structure and growth of two threatened cyprinid fishes
Source: PLoS One. 2017 Apr 17;12(4):e0175932. doi: 10.1371/journal.pone.0175932 (PMC5393870; doi:10.1371/journal.pone.0175932)
Supplement: S4 Table — The values on the diagonal are scale samples with the same age estimation for both readers. See text for statistical results. (DOCX) [file pone.0175932.s004.docx]

|  | | | | | | | | | | | |
| --- | --- | --- | --- | --- | --- | --- | --- | --- | --- | --- | --- |
| *S. laietanus* |  | |  |  | | Reader 1 | | |  |  |  |
|  | 1 | | 2 | 3 | | 4 | | 5 | 6 |  |  |
| Reader 2 | |  |  |  | |  | |  |  |  |  |
| 1 | 4 | | 1 | - | | - | | - | - |  |  |
| 2 | - | | 8 | 5 | | - | | - | - |  |  |
| 3 | - | | 2 | 15 | | 3 | | - | - |  |  |
| 4 | - | | - | 1 | | 5 | | - | - |  |  |
| 5 | - | | - | 1 | | 1 | | 3 | - |  |  |
| 6 | - | | - | - | | - | | - | - |  |  |
| 7 | - | | - | - | | - | | - | - |  |  |
| 8 | - | | - | - | | - | | - | 1 |  |  |
|  |  | |  |  |  | | |  |  |  |  |
| *B. meridionalis* | | Reader 2 | | | | | | | | |  |
|  | 0 | | 1 | 2 | | | 3 | 4 | 5 |  |  |
| Reader 2 |  | |  |  | | |  |  |  |  |  |
| 0 | 1 | | - | 1 | | | - | - | - |  |  |
| 1 | - | | 6 | - | | | 1 | - | - |  |  |
| 2 | - | | - | 9 | | | 6 | - | - |  |  |
| 3 | - | | 1 | - | | | 10 | 4 | - |  |  |
| 4 | - | | - | - | | | 1 | 7 | - |  |  |
| 5 | - | | - | - | | | - | - | 2 |  |  |
