# Supplementary figures and images for: Water abstraction affects abundance, size-structure and growth of two threatened cyprinid fishes
Source: PLoS One. 2017 Apr 17;12(4):e0175932. doi: 10.1371/journal.pone.0175932 (PMC5393870; doi:10.1371/journal.pone.0175932)

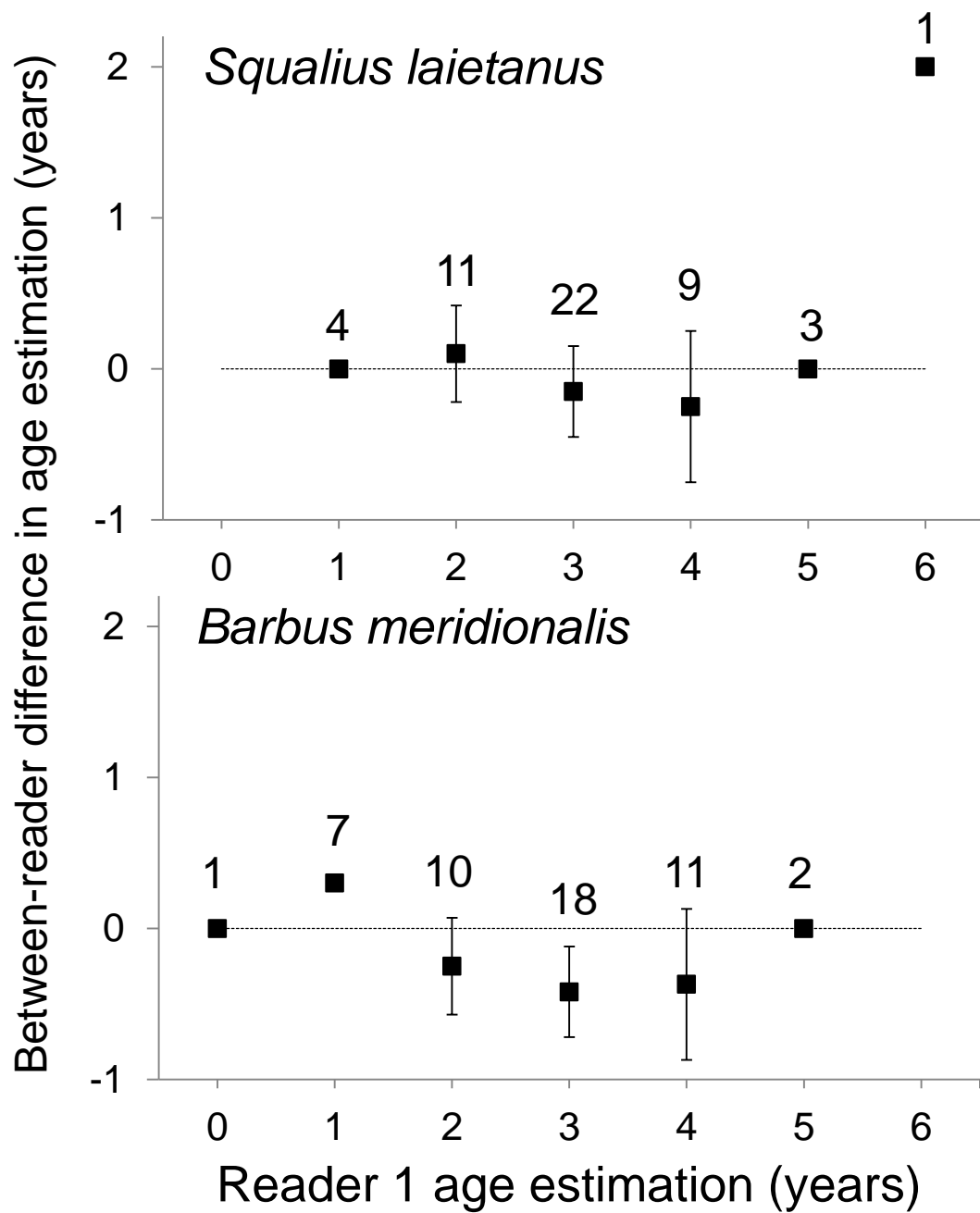

Supplement: S1 Fig — Numbers above error bars are the number of fish. (PDF) [file pone.0175932.s005.pdf]
